# Multi-line split DNA synthesis: a novel combinatorial method to make high quality peptide libraries

**DOI:** 10.1186/1472-6750-4-19

**Published:** 2004-09-01

**Authors:** Ichiro Tabuchi, Sayaka Soramoto, Shingo Ueno, Yuzuru Husimi

**Affiliations:** 1 Tokyo Evolution Research Center, 1-1-45-504, Okubo, Shinjuku-ku, Tokyo 169-0072, Japan; 2 Department of Functional Materials Science, Saitama University,255 Shimo-Okubo, Saitama 338-8570, Japan

## Abstract

**Background:**

We developed a method to make a various high quality random peptide libraries for evolutionary protein engineering based on a combinatorial DNA synthesis.

**Results:**

A split synthesis in codon units was performed with mixtures of bases optimally designed by using a Genetic Algorithm program. It required only standard DNA synthetic reagents and standard DNA synthesizers in three lines. This multi-line split DNA synthesis (MLSDS) is simply realized by adding a mix-and-split process to normal DNA synthesis protocol. Superiority of MLSDS method over other methods was shown. We demonstrated the synthesis of oligonucleotide libraries with 10^16 ^diversity, and the construction of a library with random sequence coding 120 amino acids containing few stop codons.

**Conclusions:**

Owing to the flexibility of the MLSDS method, it will be able to design various "rational" libraries by using bioinformatics databases.

## Background

The combinatorial synthesis method has been demonstrating its effectiveness in discovering novel functional molecules. Examples of this method in the field of evolutionary protein engineering are selections of a novel functional peptide from a random library on solid support [1], phage display [2] or *in vitro *virus (synonym for RNA-peptide fusion or mRNA-display) [3-5]. The efficiency of the methods depends on the screening technique employed and the library quality. In the display methods, a library of polynucleotide templates must be prepared in order to obtain a random peptide library. A primitive random library of such templates is (NNN)_n _(N = equimolar mixture of A, T, G and C). This library leads to premature short peptides and a particular bias of the amino acid composition, which makes the effective searchable sequence space biased. A slightly improved library NNK or NNS (K / S = equimolar mixture of T and C / G and C) has been conventionally used. Several methods have been developed for a more improved library. Various "rational" libraries in which the nucleotide mixtures were optimized for a target amino acid composition by using a computer calculation have been developed [6-8].

Removal of stop codons to obtain long ORFs is important for the evolutionary design of a novel protein starting from a random library. Several methods based on random block-ligation were reported [9,10]. Two high quality libraries that lead to the successful evolutionary protein design were as follows: the trinucleotide phosphoramidites (3NPs) method using twenty pre-synthesized trimers of nucleotide phosphoramidites [11-14], and the pre-selecting method using an mRNA display with a C-terminus affinity tag in order to remove stop codons [15].

We report in this article on a convenient method for the construction of a high quality library based on combinatorial DNA synthesis. This library has few stop codon and has an optimized amino acid composition for various purposes. A random library based on the split synthesis [1] is made routinely in combinatorial chemistry, but a few methods [16,17] and a few applications [18,19] have been reported for oligonucleotides synthesis. They were used for mutagenesis and the products did not have high quality for evolutionary protein engineering. We applied the split synthesis to oligodeoxyribonucleotide synthesis and developed a new procedure, based on the synthesis of designed codon mixtures using multi-line DNA synthesizers. Our method, Multi-Line Split DNA Synthesis (MLSDS), requires only standard reagents and three or four synthesizers for DNA synthesis. MLSDS can make various "rational" libraries of huge diversity with few stop codons.

## Results and Discussions

### Adaptive design to the target amino acid composition

**Figure 1:**
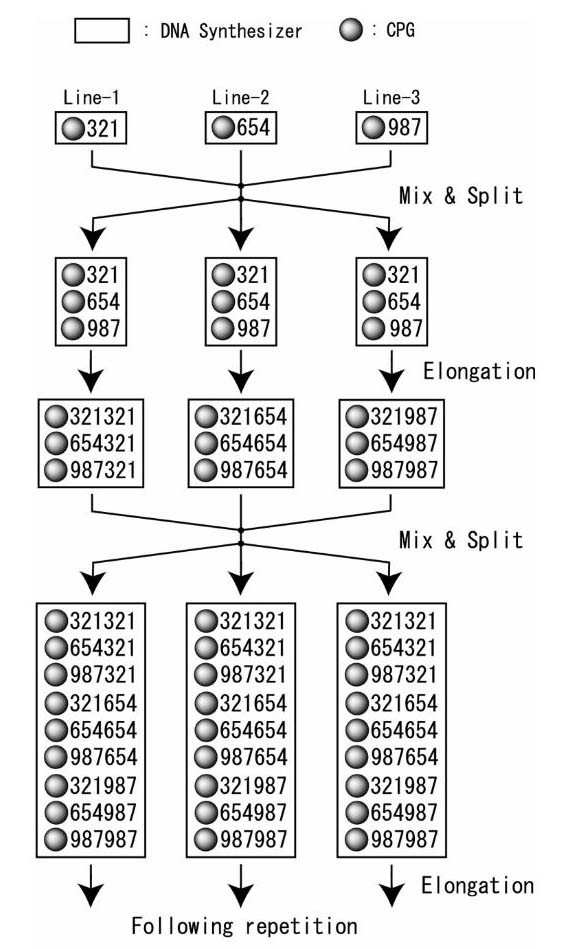
**Scheme of the MLSDS method. **The case of three-lines is shown. Uppermost three boxes indicate the state in the DNA synthesizers after the first three synthetic cycles, that is, partial mixtures of triplet codons attached to the CPGs. **1, 2 **and **3 **denote 1^st^, 2^nd ^and 3^rd ^letter mixture in the line-1 DNA synthesizer, respectively. Their A:T:G:C mixing ratios are designed with the GA program so that **123 **gives a partial mixture of triplet codons without any stop codons. In the same way, **456 **and **789 **denote corresponding partial mixtures of triplet codons in the line-2 and the line-3 DNA synthesizers, respectively. The equimolar mixture of **123, 456 **and **789**, which is obtained after split-and-mix procedure, can be designed to give approximately the target amino acid composition without stop codons. Examples of **1,2,.....,9 **are shown in Table 1.

Scheme of the MLSDS method is shown in Fig. 1 and Table 1, and described in detail in Methods section. MLSDS is able to remove not only stop codons but also particular codons. It is able to design the codon composition. We incorporated the effect of the single nucleotide deletion during a general oligonucleotide synthesis [20] into the design.

Designed biased libraries are useful for creating various novel proteins such as a functional peptide without Cys [21] or an engineered protein without Met [22]. Unnatural codons and unnatural amino acid [23] will be also incorporated in desired composition. It will be able to incorporate various results of analysis of bioinformatics databases in order to make an initial library with higher evolvability in experimental protein evolution. The optimum amino acid composition in the library may be different for each target protein. For example, when we want to explore the global protein sequence space exhaustively, the uniform amino acid composition may be the best. When we want to explore only a proven region in the protein sequence space, the use of the average amino acid composition among natural proteins [24] might be better for many aspects. When we want to design a protein with some specific properties, a library with increased or decreased fraction of specific amino acid should be constructed for each segmental region on the polypeptide chain.

Among these wide spectra of requirements, we designed DNA libraries that code peptide libraries having various characteristics and have no stop codons. Examples are: a library with the average amino acid composition of natural proteins [24], which is named "Natural" library in this article, the uniform amino acid composition; and the uniform composition except [Cys] = 0. A library encoding only four kinds of amino acid (a c-Fos mutant library [26]) was also designed. Designed molar mixing ratios of A:T:G:C for some of these libraries are shown in Table 1. Another interesting example was obtained when the target composition was "Uniform except [Met] = 0 and [Term] = 0". The designed molar mixing ratio of A:T:G:C gave the high fitness *F *value (0.96 on three lines splits) and gave no stop codon even if the effect of a point deletion was included in the GA calculation. A Met-less random library may be the best starting library for global search of the protein sequence space. This speculation is supported by the report [22] stating that a mutant dihydrofolate reductase generated by the replacement of all Met had much higher enzymatic activity than the wild type.

Internal deletion problem in the oligonucleotides synthesis process is important. It destroys the codon-based design, leading to stop-codon generation and undesirable amino acid composition. Our program incorporated deletion effects into the GA calculation and succeeded to minimize the deletion problem. Moreover it was reported that contamination of deletion products could be decreased on a denaturing PAGE for DNA of this length [15].

**Figure 2:**
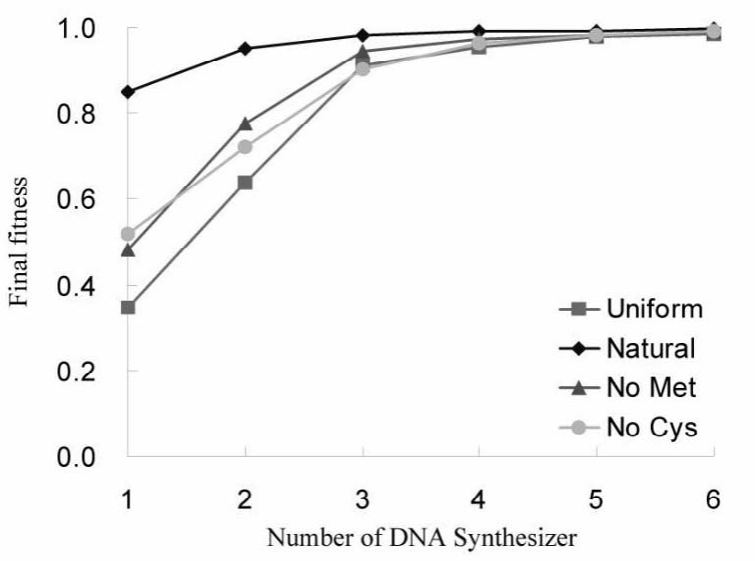
**Dependence of the final fitness on the number of DNA synthesizers for various libraries. **Ordinate: final fitness (=correlation coefficient). Abscissa: line number of DNA synthesizers. Libraries are "Uniform", "Natural", "No Met" and "No Cys".

We also investigated the practical number of DNA synthesizers. For this purpose, we calculated the final correlation coefficient between the designed and the various target compositions with up to 6-line DNA synthesizers. As shown in Fig. 2, the final correlation coefficient (= the final fitness) became saturated at about 3- or 4-lines on this program. Our GA program is not the best for obtain best *F *value but suitable for designing actual synthesizing operations. These results showed MLSDS method gave a high quality library even with three DNA synthesizers.

When we took the natural abundance as the target amino acid composition, we got a highest fitness value *F *= 0.99 (on three lines) in the GA calculations. This is reasonable, because the average amino acid composition among natural proteins highly correlates to the number of synonymous codons in the standard genetic code table [25].

### Synthesis of MLSDS libraries

We synthesized a "Natural" library and a "Uniform except [Cys] = 0" library mentioned in the previous section. In Table 2 the compositions of the actually synthesized DNA libraries are listed in comparison with the target compositions. They were high quality libraries (*F *= 0.85 and 0.66, respectively) without stop codons in full-length DNAs. The deletion rate was about 0.3% per coupling. For the total DNAs including deletants, *F *= 0.90 and 0.60, respectively.

We also synthesized MLSDS products composed of limited kinds of amino acid. It has been regarded that such a peptide can be synthesized only by 3NP method. A mutant c-Fos library that contained only four kinds of amino acid was synthesized, which was equivalent to a library synthesized by 3NP method [26]. It was a high quality library (*F *= 1.00) (Table 2). So far, fifteen libraries with various amino acid compositions were successfully synthesized.

**Figure 3:**
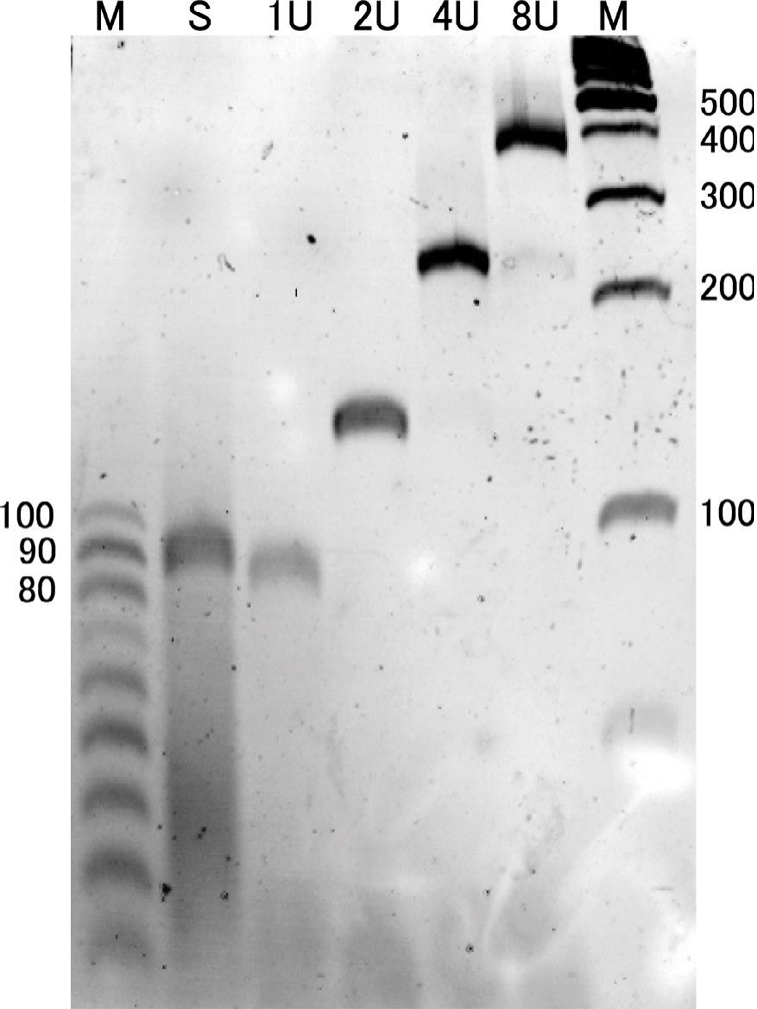
**Production of 2×, 4×, and 8× length library. **Lane-M: Size marker. 10 base ladder (left) and 100 base ladder (right). Lane-S: Initial synthesized library (87 mer). Lane-1U: Initial single-unit library (94 mer) made by PCR amplification. Lane-2U: Double-unit library (138 mer) made by ligation product of digested 1U. Lane-4U: quadruple-unit library (240 mer) Lane-8U: octuple-unit library (444 mer). PAGE was done with 5 % polyacrylamide, TAE buffer (pH 8.0), 8 M urea, 65°C and stained with SybrGreen I and visualized with a fluorescence imager (Bio-Rad FX).

In order to make long ORFs, we assembled 8 units of the oligomers. Stem sequences of them did not have any stop codons. A DNA library encoding 120 amino acids plus nine 5'- and 3'-flanking semi-random di-peptides (thus, total 138 amino acids) was constructed (Fig. 3).

The diversity of the synthesized library is about 10^16 ^judging from the mass (data of A_260_) and purity (data of PAGE) of synthesized DNA. With an *in vivo *selection, there is a diversity limit by the transformation step. But with an *in vitro *selection, there is no such limitation. Thus exploration of huge sequence space by *in vitro *virus [3-5] or related techniques [28,29] will become possible, depending on the experimental cost.

### Comparison of MLSDS with other methods

So far, a really random library has been generated by four methods. Other methods do not give a really random library, because they can not provide a library in which all the 20 amino acids are encoded at all sites. A comparison of library quality for three methods is shown in Table 3.

An application of 3NPs method to mutagenesis of antibodies [27] or coiled-coils [30] gave good results. Twenty kinds of 3NPs mean one codon per one amino acid, but the codons are degenerate. Thus 3NPs method makes many tRNAs useless. The translation efficiency was calculated based on the codon usage, giving maximum 4-fold decrease in *Triticum aestivum*. It was reported that the reaction efficiency of 3NPs was far from uniform. The sequence data of synthesized DNA using an equimolar mixture of 19 kinds of 3NPs (without Cys) showed 12-fold (maximum) difference in composition [27] or more [12]. The coupling yield was affected by the mixing ratio of 3NPs and by the context, showing 8-fold (maximum) difference for the same 3NP [27]. Thus it will be difficult to correct reaction efficiencies by adjusting the mixing ratio. The correlation coefficient between the target composition and the actual composition was about 0.4 (for uniform 19 kinds of amino acids) [27] (Table 3). Dimer-phosphoramidites [17] method is a variation of 3NPs method, using pre-synthesized amidites, and had the same problems. In fact, the bias was observed [17].

A pre-selecting method using an mRNA display [15] was fruitful in evolutionary protein design. Novel peptide aptamers were evolved starting from a long ORF random library [31,32]. But this method could not remove all the stop codons. It gave limited library diversity. This method has low flexibility in amino acid composition. For example it is difficult to generate a "Uniform except [Met] = 0" library. The correlation coefficient between the target composition and the actual composition were not so high (Table 3).

The Y-Ligation Block Shuffling (YLBS) method [9] has high potentiality in the evolutionary design of peptides. It has problems on deletion and reaction bias of RNA ligase.

MLSDS produced libraries with high quality as shown in Table 3. Above-mentioned problems are not so severe for MLSDS method, because it uses only standard phosphoramidites and is free from any biochemical bias such as in mRNA display and in YLBS. It was reported that the difference in the reaction efficiency of equimolar mixture of four kinds of mono-phosphoramidites was only about 1–5 % [33,34]. MLSDS can create any specific amino acid composition as same as 3NP method, and a MLSDS library is made at lower cost than that made with other methods.

## Conclusions

We applied the split synthesis to oligodeoxyribonucleotide synthesis and developed a new procedure, Multi-Line Split DNA Synthesis (MLSDS), based on the synthesis of designed codon mixtures using three-line DNA synthesizers. MLSDS can make various "rational" libraries of huge diversity with few stop codons by using bioinformatics databases. Combination of an MLSDS library with a screening method for huge diversity will accelerate the protein evolution *in vitro*.

## Methods

A random MLSDS library was synthesized as follows. A standard DNA synthesis method was used in three lines of DNA synthesizer running in parallel. The randomized regions were combinatorialy synthesized in codon units. Triplet codons were synthesized separately in the three synthesizers as an elongation reaction of oligonucleotides on beads made of controlled pore glass (CPG). CPG beads were mixed together manually, and then splitted again into three reaction tubes manually and the next triplet codons were synthesized (Fig. 1).

The sequence of a 87 mer library was 5'-GAT GAG GCG AAG ACG *NAC TGS *(***123/456/789***)_15 _*NAC TGS *GAG GCT GGC TGC CAC-3', where N and S denote A/T/G/C and G/C, respectively. The A:T:G:C mixing ratio in each letter of three codon groups **123**, **456**, and **789 **was shown in Table 1. These values were calculated as described below. Both flanking regions contain the recognition sequences of type-IIs restriction enzymes *Bbs*I and *Bbv*I, respectively. In order to make longer sequences, we ligated 2 to 8 units of oligomers at the cohesive ends (the underlined sequences shown above) generated by the restriction enzyme treatment. The assembly method was as described in Ref. [16]. The italicized sequence shown above represents the assembly unit (random region of 45 bp and flanking semi-random linking region of (6+6)/2 bp).

The synthesized DNA libraries were amplified by PCR using KOD Dash polymerase (TOYOBO), inserted into pCR2.1TOPO vector (Invitrogen) and cloned, avoiding cloning bias. The clones were sequenced.

Computer calculations to determine the optimum molar mixing ratio of four bases in the codon synthesis step were performed by using Mathematica (Wolfram Research). We made a GA program for this purpose. Firstly, the target amino acid composition, **p**_*T *_= (*p*_*T*1_*, *p_*T*2_*, p*_*T*3_,..., *p*_*T*21_), was established, where normally *p*_*T*21 _= 0 for stop codons. Secondly, we calculated an expected amino acid (plus stop codons) composition, **p **= (*p*_1_, *p*_2_, *p*_3_,.., *p*_21_), from the molar mixing ratio of the bases, ***x ***= (*x*_1_, *x*_2_, *x*_3_,....., *x*_12*L*_), where 12*L *is equal to 4(number of bases) × 3(number of codon letters) × *L*(number of synthesizer-line). For example, the mixtures for the first letter and the second letter of the first DNA synthesizer have the molar mixing ratio [A]: [T]: [G]: [C] = *x*_1 _: *x*_2 _: *x*_3 _: *x*_4 _and *x*_5 _: *x*_6 _: *x*_7 _: *x*_8_, respectively. And for example, when *L *= 3, the expected alanine composition *p*_1 _is given by:


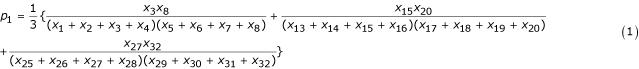


for the full-length sequence without deletion.

We solved an integer-programming problem (6-valued 12*L*-dimensional optimization problem) having the solution *x*_*i *_as integer (0,1,2,3,4,5). The reason for 6-digits "integer" was to simplify the DNA synthesizer handling and also to simplify the calculation. As the fitness *F *of ***x ***in the GA, we took a correlation coefficient between the expected (or designed) amino acid composition and the target amino acid composition:


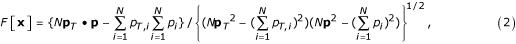


where *N *= 21 for our normal case. The optimum ***x***, which gave the maximum fitness *F*, was calculated using a simple GA program.

It was reported the deletion rate during a general oligonucleotide synthesis is about 0.5% per coupling [20], and our data (about 0.3% per coupling) were compatible with this value. We incorporated the effect of the single nucleotide deletion into the GA calculation. We considered only the affect of a point deletion in a synthesized oligonucleotide because the deletion rate is low enough. When a point deletion occurs in the 5' constant region, all the amino acids in the random region are the frame shifted ones. When the event occurs at the i-th site of the random region, it affects the composition in the all downstream from the i-th site, and so on. We incorporated all these effects into the calculation of the composition. Details are described in Additional file 1.

## Authors' contributions

IT conceived of this specific study and participated in its design and coordination. SS carried out GA calculation. SU carried out the sequence analysis and the making of the assembled longer sequence. YH conceived of general background and mathematical detail.

## Abbreviations

MLSDS, multi-line split DNA synthesis; ORF, open reading frame; CPG, controlled pore glass; PAGE, polyacrylamide gel electrophoresis; GA, genetic algorithm; 3NPs, trinucleotide phosphoramidites.

## Supplementary Material

Additional File 1 In the additional WORD file (MLSDS22AdditionalFile.doc), the detail of calculation method is described for the the expected amino acids composition **p **= (*p*_Ala_, *p*_Arg_, ....., *p*_term_) considering the single nucleotide deletion. Click here for file

## Figures and Tables

**Table 1:** Examples of molar mixing ratio of bases for MLSDS libraries

**Library name**	**No Cys**									**Natural**								
**Line Number of Synthesizers**	Line-1			Line-2			Line-3			Line-1			Line-2			Line-3		

**ID Number of premix phosphoramidites**	**1**	**2**	**3**	**4**	**5**	**6**	**7**	**8**	**9**	**1**	**2**	**3**	**4**	**5**	**6**	**7**	**8**	**9**
**Molar mixing ratio of A:T:G:C**																		
						
**A**	2	3	5	5	0	0	2	5	0	5	4	2	2	0	0	3	4	0
**T**	0	0	3	4	3	0	3	5	2	0	1	1	3	2	3	4	2	5
**G**	3	0	0	4	3	1	1	0	0	5	0	2	5	5	2	4	0	0
**C**	3	1	0	0	1	0	1	3	5	3	1	0	2	2	1	2	4	0

**Table 2:** Comparison of the amino acid compositions of the actually synthesized DNA libraries (the full length library) with the target composition

**Library name**	**Natural**		**No Cys**		**c-Fos e', g'-**	
			
	Target	Actual	Target	Actual	Target	Actual
Ala	8.9%	11.4%	5.3%	6.7%	0.0%	0.0%
Arg	4.7	3.5	5.3	5.9	30.0	29.7
Asn	4.4	3.5	5.3	4.3	0.0	0.0
Asp	5.5	8.6	5.3	5.9	0.0	0.0
Cys	2.8	2.5	**0.0**	**0.0**	0.0	0.0
Gln	3.9	2.1	5.3	5.5	20.0	19.6
Glu	6.2	6.9	5.3	5.5	20.0	25.0
Gly	7.8	12.0	5.3	8.2	0.0	0.0
His	2.0	1.2	5.3	2.7	0.0	0.0
Ile	4.6	3.9	5.3	5.9	0.0	0.0
Leu	7.5	3.7	5.3	4.3	0.0	0.0
Lys	7.0	6.2	5.3	4.7	30.0	27.9
Met	1.7	1.1	5.3	3.5	0.0	0.0
Phe	3.5	4.0	5.3	5.1	0.0	0.0
Pro	4.6	2.5	5.3	2.4	0.0	0.0
Ser	7.1	8.1	5.3	1.6	0.0	0.0
Thr	6.0	5.8	5.3	6.7	0.0	0.0
Trp	1.1	1.8	5.3	8.2	0.0	0.0
Tyr	3.5	4.4	5.3	4.7	0.0	0.0
Val	6.9	6.9	5.3	8.2	0.0	0.0
Term	**0.0**	**0.0**	**0.0**	**0.0**	**0.0**	**0.0**
Number of sequenced codon	-	568	-	255	-	219
Correlation coefficient	-	0.85	-	0.66	-	1.00
Sum of absolute errors	-	26.9%	-	28.1%	-	5.66%

"Term" denotes stop codons. In each target composition, [Term] = 0. "Natural": the library with the natural abundance (the average composition in 207 natural proteins) [18]; "No Cys": the library with uniform composition except [Cys] = 0; "c-Fos e', g'-": a c-Fos mutant library containing only 4 kinds of amino acid as same as in Ref.[26].

**Table 3:** The comparison between the target amino acids composition and the actual composition of various libraries.

A. Uniform Type			
Method	3NPs	mRNA display	MLSDS

Reference, Library	[29], 96T λ 6	[18], Random	This work, No Cys
Target value	Equimolar 19 amino acids	Equimolar 20 amino acids	Equimolar 19 amino acids
Correlation coefficient ^a^	0.43	0.56	0.66
Sum of absolute errors ^b ^[%]	54.5	23.7	28.1
Percentage of stop codon	ND ^c^	0.46	0.00
Percentage of cassettes containing stop codon	ND ^c^	8.33	0.00


B. Doping Type			

Method	3NPs	mRNA display	MLSDS

Reference, Library	[29], 100z	[18], β-Cassette polar	This work, c-Fos, e', g'-
Target value	Biased	Polar amino acid	Biased
Correlation coefficient ^a^	0.94	0.56	1.00
Sum of absolute errors ^b ^[%]	42.4	73.7	5.66
Percentage of stop codon	ND ^c^	0.39	0.00
Percentage of cassettes containing stop codon	ND ^c^	6.25	0.00

The values were calculated using the data of full-length (without deletion) libraries. Values of 3NPs and mRNA display method were calculated with some assumptions. In mRNA display method, the value of stop codons were estimated from data described in the article and some assumptions; one cassette did not have two or more stop codons and stop codons appeared equally in every position. The target composition of β-Cassette polar is assumed that polar amino acids are equi-molar and the others are 0%.

^a^Correlation coefficient is calculated using target and actual amio acids composition including stop codons contribution. And it is assumed that stop codons were not appeared in full-length libraries of 3NPs method.

^b ^Sum of absolute values of difference between each target and actual amino acid.

^c ^ND is abbreviation of no data.
