# Supplementary material for: Multi-line split DNA synthesis: a novel combinatorial method to make high quality peptide libraries
Source: BMC Biotechnol. 2004 Sep 1;4:19. doi: 10.1186/1472-6750-4-19 (PMC520752; doi:10.1186/1472-6750-4-19)
Supplement: Additional File 1 — In the additional WORD file (MLSDS22AdditionalFile.doc), the detail of calculation method is described for the the expected amino acids composition p = (pAla, pArg, ....., pterm) considering the single nucleotide deletion. [file 1472-6750-4-19-S1.doc]

**Appendix:** Formula for the expected amino acids composition **p** = (*p*_Ala_, *p*_Arg_, ….., *p*_term_) considering the single nucleotide deletion.

We consider a synthesized DNA, which has 5’-constant region of *n* base long, a random sequence region of *m* base long and a 3’-constant region. We assume that the probability of single base deletion at any site *i* is *a* (independent of *i*). In practice, *a* is much less than unity, we neglect multiple deletions in the same strand.

The amino acid composition vectors **c**_f_, **d**_1_, **d**_2_, **d**_3_, **e**_1_, **e**_2_, and **e**_3_ defined below can be calculated from the mixing ratio ***x*** =(*x*_1_, *x*_2_, *x*_3_,….,*x_1_*_2_*_L_*) defined in Materials & Methods.

**c***_f_* : expected amino acid composition when no deletion occurs. An example of an element is given by Eq.(1) in the text.

**d***_1_* : expected amino acid composition in the frame-shifted region.

**d***_2_* : expected amino acid composition for codons with a deletion at the 2^nd^ letter.

**d***_3_* : same as above except at the 3^rd^ letter

**e***_1_* : expected amino acid composition for the last codon in the random region with a deletion at the 1^st^ letter.

**e***_2_* : same as above except at the 2^nd^ letter

**e***_3_* : same as above except at the 3^rd^ letter

We introduce following parameters:

$$Z=\left[\frac{i}{3}\right]$$

$$R=\frac{m}{3}$$

$$Y=\left[\frac{i-n-1}{3}\right]$$

where [*s*] denotes Gauss’s symbol, that is, the maximum integer less than *s*.

Finally, we get the formula for the expected amino acid composition **p** as a function ***x***, *a, n*, and *m*:

$\begin{matrix} \mathbf{Q}(\mathbf{x},a,n,m)\equiv\\ \sum_{i=n-1}^{n+m-3} \frac{a(1-a)^{i-1}}{R-1}\left(Y\mathbf{c}_{f}+\left(R-Y-2\right)\mathbf{d}_{1}-\left(i-3Z-2\right)\left(i-3Z\right)\mathbf{d}_{1}+\frac{1}{2}\left(i-3Z-1\right)\left(i-3Z\right)\mathbf{d}_{2}+\frac{1}{2}\left(i-3Z-1\right)\left(i-3Z-2\right)\mathbf{d}_{3}\right) \\ \\ \mathbf{p}(\mathbf{x},a,n,m)=\left(1-a\right)^{n+m-1}\mathbf{c}_{f}+\left(1-\left(1-a\right)^{n-1}\right)\mathbf{d}_{1}+\mathbf{Q}(\mathbf{x},a,n,m)+a\left(1-a\right)^{n+m-4}\mathbf{e}_{1}+a\left(1-a\right)^{n+m-3}\mathbf{e}_{2}+a\left(1-a\right)^{n+m-2}\mathbf{e}_{3} \end{matrix}$ (A1)

As expected from the definition, when *a* is around 0.005 and *m* is around 50, we can neglect terms concerning **d**_2_, **d**_3_, **e**_1_, **e**_2_, and **e**_3_. We incorporated the effect of the single nucleotide deletion into the GA calculation by using Eq.(A1) instead of Eq.(1) in the text.
